# Early arteriovenous malformation mimicking pediatric capillary malformation: diagnostic value of infrared thermography

**DOI:** 10.1186/s41065-025-00484-7

**Published:** 2025-06-23

**Authors:** Hao Gu, Xiaojie Yue, Xiong Zhao, Qiang Shu

**Affiliations:** 1 Department of Burn and Plastic Surgery, Multidisciplinary Team of Vascular Anomalies, The Children’s Hospital, Zhejiang University School of Medicine, National Clinical Research Center for Child Health, Hangzhou, PR China; 2 Department of Cardiac Surgery, The Children’s Hospital, School of Medicine, Zhejiang University, National Clinical Research Center for Child Health, Hangzhou, PR China

**Keywords:** Capillary malformation, Arteriovenous malformation, Infrared thermography, Differential diagnosis, Port wine stain

## Abstract

Early-stage arteriovenous malformations (AVMs) and port-wine stains (PWS) exhibit overlapping clinical presentations, notably as flat, irregular erythema, posing significant diagnostic challenges. Conventional imaging techniques offer insufficient resolution for microvascular assessment during initial evaluation, and definitive diagnosis requires invasive tissue biopsy. We report a case of a 4-year-old boy initially misdiagnosed with facial PWS following ineffective photodynamic therapy. Digital images with quantitive readings acquired by infrared thermography (IRT) camera detected localized hyperthermia within the lesion, indicative of underlying hemodynamic anomalies. Skin biopsy of the lesion was performed and a somatic *KARS* mutation (p. Gln61His) was detected via targeted sequencing. Subsequent digital subtraction angiography confirmed the presence of micro-arteriovenous fistulas, thereby confirming the diagnosis of early AVM. This case illustrates the clinical utility of IRT in detecting subtle temperature variations associated with vascular pathophysiology. Integrating IRT into initial diagnostic algorithms may enhance the accuracy of differential diagnosis for vascular malformations, particularly early-stage AVMs.

## Introduction

Capillary malformations (CMs), also known as port wine stains (PWS), are congenital, progressive low-flow vascular anomalies affecting cutaneous tissues, with an estimated incidence of 0.3–0.5% in live births [1]. PWS typically present as sporadic, unifocal, flat, irregular cutaneous patches exhibiting from pink to purple [2]. In comparison, arteriovenous malformations (AVMs) represent high-flow anomalies characterized by aberrant direct connections between arterial and venous systems. Early-stage AVMs often demonstrate clinical manifestations indistinguishable from PWS; however, their natural progression can lead to significant morbidity, including pain, hemorrhage, ulceration, functional impairment of affected tissues, and high-output cardiac failure [3–5]. Facial AVMs may initially present with cutaneous lesions mimicking PWS, posing substantial diagnostic challenges during initial evaluation. Doppler ultrasound (US) is frequently employed to differentiate CMs from AVMs, but its diagnostic accuracy is operator-dependent and limited in resolving microvascular architecture [6]. In genetic perspective, PWS is primarily driven by somatic *GNAQ* mutations, whereas AVMs involve pathogenic variants in the Ras/Raf/MAPK signaling pathway [7–9]. In general, the detection of somatic mutations currently necessitates invasive tissue biopsy. Consequently, accurate discrimination between AVMs and suspected PWS during preliminary assessment is clinically imperative. Herein, we present a special pediatric case of unilateral pediatric “capillary malformation” that was ultimately diagnosed as AVM through combination of genetic sequencing and infrared thermography (IRT), underscoring heightened awareness of differentiating early-stage AVMs and application of IRT in the diagnostic workflow during the initial evaluation of presumed pediatric skin erythema.

## Case report

**Fig. 1:**
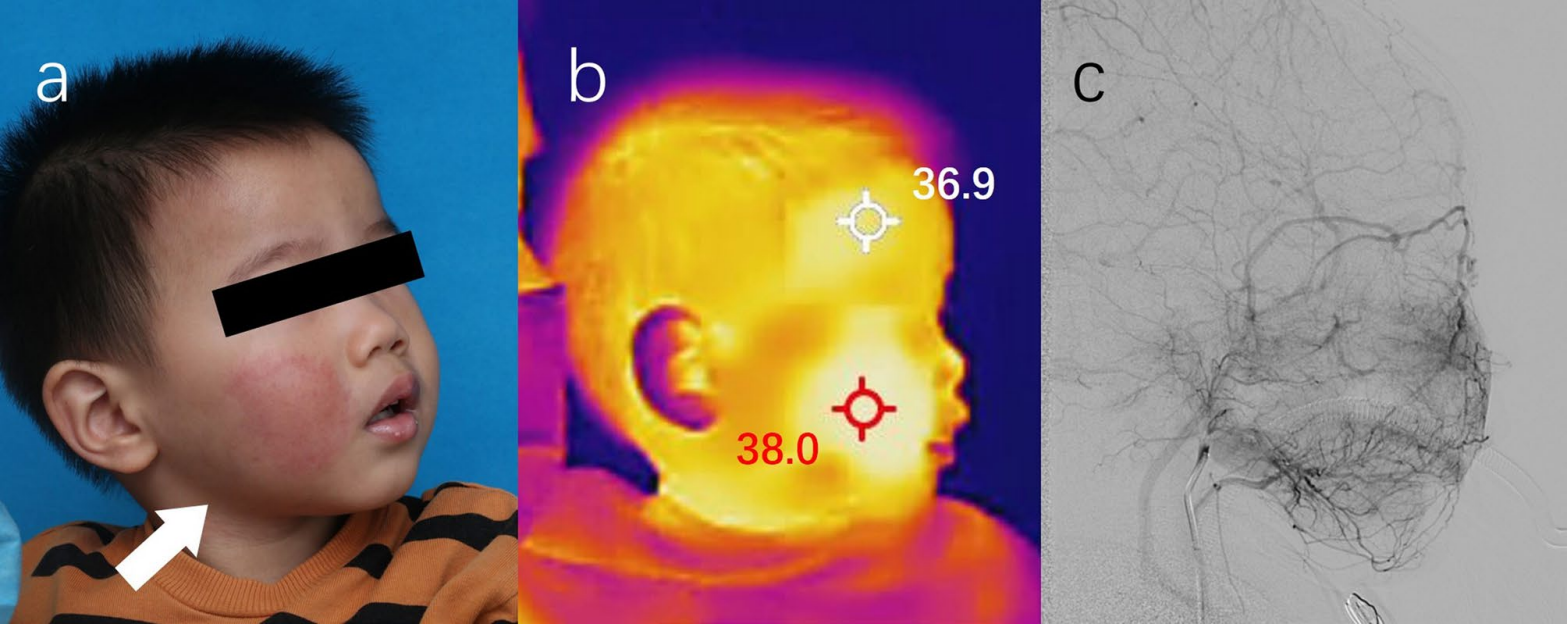
**1a**. Skin erythema located in the buccal region of right face (white arrow). **1b**. Localized hyperthermia detected by infrared thermography in the buccal lesion. **1c**. Micro-arteriovenous fistula observed in right buccal lesion by digital subtraction angiography

A 4-year-old male presented with unilateral facial erythema involving the right buccal region (Fig. 1a). He was born at 38 weeks to a 32-year-old mother via a eutocia. The skin lesion of erythema was noted instantly after the birth and no other cutaneous involvement was observed. The erythema remained stable but became subjectively warmer during physical activity or emotional distress. Family history was negative for hereditary vascular anomalies or cutaneous birthmarks on both parental sides.

The patient had previously received a diagnosis of port-wine stain at another medical institution and underwent multiple photodynamic therapy (PDT) sessions without observable clinical improvement. Physical examination revealed mild relative hyperthermia within the lesional tissue compared to contralateral and adjacent areas. Ophthalmologic and neurologic assessments showed no evidence of glaucoma or seizure predisposition. Doppler US demonstrated no detectable vascular flow within the lesion. Then, a portable IRT camera (Hikmicro W-Pocket2, Hikvision Digital Technology Company Ltd., Hangzhou, China) quantitively converted infrared energy into digital images (namely thermo-grams). The difference of temperature in Centigrade between the lesion and adjacent normal skin was displayed on the grams by thermal readings of comparable significance. Subsequently, the thermo-grams confirmed regional temperature elevation of 1.1 ℃ in the affected area (Fig. 1b) and guided the skin biopsy site selection. Minimally invasive needle biopsy enabled paired tissue and blood analysis via targeted next-generation sequencing (NGS), identifying a somatic *KARS* variant (p. Gln61His) at 1.5% allele frequency (read depth: 13,358×). Digital subtraction angiography (DSA) subsequently revealed micro-arteriovenous fistulae (Fig. 1c). Integration of genetic and radiologic evidence established a definitive diagnosis of early-stage facial arteriovenous malformation.

## Discussion

Arteriovenous malformations, a kind of rare but intractable high flow vascular malformations characterized by aberrant artery-venous shunting, lead to progressive complications including tissue damage, hemorrhagic risks, and substantial morbidity [10]. The present case highlights a critical diagnostic dilemma: facial AVMs frequently mimic port-wine stains (PWS) in their early stage as flat erythematous lesions, despite divergent therapeutic requirements and prognostic trajectories. This morphological overlap necessitates advanced diagnostic modalities to guide clinical decision-making.

Magnetic resonance imaging (MRI) is the most widely-recognized imaging technique for vascular malformations assessment, with multiparametric protocols (e.g., T2-weighted fat-suppressed sequences, contrast-enhanced MR angiography) enabling precise delineation of lesion architecture, perfusion characteristics, and anatomical relationships [11, 12]. However, MRI demonstrates limited sensitivity for superficial cutaneous lesions confined to skin/subcutaneous layers. While Doppler ultrasonography remains the primary screening tool due to its real-time accessibility and absence of radiation [13], its diagnostic accuracy depends heavily on operator expertise and patient compliance—a significant limitation in pediatric populations. Histopathological analysis remains the diagnostic gold standard for vascular anomalies when imaging yields equivocal results, necessitating tissue biopsy for definitive classification and genetic interpretation [14].

Thermal heterogeneity emerges as a key distinguishing feature between high-flow AVMs and low-flow PWS [15, 16]. Elevated cutaneous temperature correlates with augmented blood flow in AVMs, serving as a surrogate marker for underlying hemodynamic disturbances [15]. Conventional palpation-based temperature assessment appears subjective and non-quantitative. IRT addresses these limitations by providing spatial thermal mapping with 0.05℃ resolution [17]. Our case corroborates recent evidence of IRT’s diagnostic utility in vascular anomalies, including infantile hemangiomas [18]. In the evaluation process, IRT identified localized hyperthermia in the buccal regions that subtly supported our distinguishment and directed subsequent genetic and angiographic confirmation of AVM, despite inconclusive US.

Nevertheless, the limitations of this brief report warrant consideration. The sample size in a single pediatric center, while adequate for preliminary findings, may not reflect the broader age spectrum of vascular malformations. Additionally, while IRT demonstrated superior sensitivity to ultrasound in our case, its specificity relative to established imaging modalities, such as MRI, remained verified through controlled comparative studies. Furthermore, ambient temperature fluctuations and emotional states introduce physiological “noise” requiring standardized imaging protocols. Future multi-institutional trials should establish diagnostic thresholds for pathologic thermal signatures and evaluate IRT’s cost-effectiveness in clinical workflows.

In conclusion, the implications of this report extend beyond merely improving diagnostic accuracy. The integration of IRT into diagnostic algorithms for cutaneous vascular anomalies represents a paradigm shift that empowers clinicians to differentiate AVMs from PWS at early stage, leading to appropriate interventions, optimal treatment response and decreased risk of AVM-related complications. As thermal imaging technology evolves, its synergistic application with genetic and radiographic modalities may redefine precision medicine in vascular anomaly care.

## Data Availability

No datasets were generated or analysed during the current study.
